# Blood-Based Oxidative Stress Markers and Cognitive Performance in Early Old Age: The HAPIEE Study

**DOI:** 10.1159/000450702

**Published:** 2016-11-02

**Authors:** Pia Horvat, Ruzena Kubinova, Andrzej Pajak, Abdonas Tamosiunas, Ben Schöttker, Hynek Pikhart, Anne Peasey, Magdalena Kozela, Eugene Jansen, Archana Singh-Manoux, Martin Bobak

**Affiliations:** ^a^Department of Epidemiology and Public Health, University College London, London, UK; ^b^National Institute of Public Health, Prague, Czech Republic; ^c^Department of Epidemiology and Population Studies, Jagellonian University Collegium Medicum, Krakow, Poland; ^d^Department of Population Studies, Institute of Cardiology, Lithuanian University of Health Sciences, Kaunas, Lithuania; ^e^Division of Clinical Epidemiology and Aging Research, German Cancer Research Center, Heidelberg, Germany; ^f^Network Aging Research, University of Heidelberg, Heidelberg, Germany; ^g^Center for Health Protection, National Institute for Public Health and the Environment, Bilthoven, The Netherlands; ^h^INSERM U 1018, Villejuif, France

**Keywords:** Oxidative stress, Free radicals, Aging, Cognitive function, Cohort studies, Epidemiology

## Abstract

**Background/Aims:**

Oxidative stress is involved in Alzheimer disease pathology, but its impact on cognitive function in community-dwelling older adults remains unknown. We estimated associations between serum oxidative stress markers and cognitive function in early old age.

**Methods:**

Subjects aged 45-69 years recruited in urban centers in Central and Eastern Europe had memory, verbal fluency, and processing speed assessed at baseline (2002-2005) and 3 years later. Derivatives of reactive oxygen metabolites (d-ROMs), biological antioxidant potential (BAP), and total thiol levels (TTLs) were measured at baseline in a subsample. Linear regression was used to estimate associations of biomarkers with cognitive test scores cross-sectionally (*n* = 4,304) and prospectively (*n* = 2,882).

**Results:**

Increased d-ROM levels were inversely associated with global cognition and verbal fluency cross-sectionally and in prospective analysis; observed effects corresponded to 3-4 years' higher age. TTL was inconsistently associated with memory. BAP was not related to cognitive function.

**Conclusion:**

This study found modest evidence for a relationship between serum d-ROMs and cognitive function in a population sample of older adults.

## Introduction

The oxidative stress/free radical theory remains one of the leading mechanistic explanations of aging [1]. Irreversible oxidative damage of cells and organs is seen to progressively accumulate as a consequence of the growing systemic imbalance between the production of reactive oxygen species (ROS) and oxygen-derived free radicals and the available antioxidant defenses. The resulting oxidative stress contributes to age-related functional decline and increased risk for a number of chronic diseases. Harman [2] first advanced the free radical theory in 1954, and recent research has extended it to work on a range of outcomes, including healthy lifespan [3].

One of the key outcomes where oxidative stress is hypothesized to play an important role is Alzheimer disease (AD) [4]. AD is the principal cause of dementia and is characterized by progressive decline in memory and other cognitive abilities [5]. Even in the absence of overt dementia, impaired cognitive status is associated with great personal and social costs [6]. Furthermore, as dementia involves changes over a long period, perhaps decades [7], there is increasing interest in the determinants of individual differences in cognitive performance in adulthood [8]. Identification of reliable oxidative stress markers which could serve as biomarkers of aging may not only increase our understanding of age-related cognitive decline but also help to identify individuals at a greater risk of future cognitive impairment.

Patients with AD and mild cognitive impairment have shown elevation of markers of lipid peroxidation and decreased total antioxidant capacity of blood, indicating an increase in oxidative stress [9]. However, this relationship has rarely been investigated in the general population. An early community study found greater subsequent cognitive decline at high levels of systemic oxidative stress as measured by thiobarbituric acid-reactive substances [10]; however, lipid peroxidation is not seen to be a universal marker of oxidative stress status [11], and the possibility of cognitive domain-specific differences in vulnerability to oxidative stress remains to be investigated. In addition, the disappointing results of clinical trials with antioxidants conflict with the studies demonstrating that different biomarkers of oxidative stress are elevated in the brain of AD patients and with epidemiologic studies which show that dietary antioxidants reduce the risk of AD, suggesting that the role of oxidative stress in AD and cognitive decline is yet to be firmly established [12].

The lack of population-based studies may partly reflect the relative difficulty and cost of measuring antioxidant potential of stored blood samples. However, the recent availability of simpler methods for detecting ROS by using derivatives of reactive oxygen metabolites (d-ROMs) and biological antioxidant potential (BAP) has led to their increasing use in large population studies [13,14,15].

Accordingly, the aim of the present study was to investigate the associations of serum oxidative stress markers (d-ROM levels, total thiol levels [TTLs], and BAP) with cognitive performance in older adults in 3 Central and Eastern European cohorts in cross-sectional and prospective analyses.

## Materials and Methods

### Study Populations and Participants

The HAPIEE (Health, Alcohol and Psychosocial Factors in Eastern Europe) study protocol has been described previously [16]. Because of legal restrictions on exporting human biological materials from Russia, biomarkers were not measured in the Novosibirsk cohort (*n* = 9,360), so only 3 of the 4 cohorts were included in the current analysis. Random samples of men and women aged 45-69 years at baseline were recruited in Krakow, Poland (*n* = 10,728), and in 6 middle-sized Czech towns (*n* = 8,857) in 2002-2005 and in Kaunas, Lithuania (*n* = 7,161), in 2006-2008. The response rates were 61% in Krakow, 65% in Kaunas, and 55% in Czech towns. Baseline data were collected by questionnaire and a short clinical examination, which included drawing a fasting venous blood sample. Czech and Krakow participants were visited by a nurse in their homes to complete the questionnaire and invited to a clinic for the examination. In Kaunas, both the questionnaire and examination were completed at a clinic. The second wave of data was collected by questionnaire in 2006-2008 in Czech towns and Krakow (this was the baseline survey in Kaunas), with an overall response rate of 61%.

At Czech and Krakow baseline, cognitive assessment (*n* = 7,975) was conducted in participants aged 60 years or over and a random sample of approximately 20% of participants younger than 60 years. In 2006-2008, cognitive assessment was completed by all Kaunas participants (*n* = 7,059) and all participants at follow-up in Czech towns and Krakow irrespective of their age (*n* = 11,832); for 57.5% (*n* = 6,801) of Czech and Polish participants, this was the first cognitive assessment. Cognitive assessment was conducted at a clinic, except for Krakow, where it took place in participants' homes.

Because of limited funding, biomarkers were analyzed in a nested case-control study. From 26,746 participants eligible for the nested case-control study, 3,462 participants were excluded a priori because they were missing a blood sample, another 1,867 had not consented to follow-up assessments, and 208 had an unconfirmed cardiovascular event. Cases (*n* = 1,882) were participants who died from any cause or experienced a nonfatal cardiovascular event (myocardial infarction [MI] or stroke) between baseline and December 31, 2010 (December 31, 2009, in Krakow). Each case was matched to at least 2 controls drawn randomly from the study population by age (in 5-year bands), sex, and study center (*n* = 4,476).

### Cognitive Assessment

Cognitive function was assessed by 4 neuropsychological tests, as described previously [17]: (1) immediate word recall (10 nouns over 3 consecutive 1-min trials; possible range 0-30); (2) delayed recall (10 nouns following an interval; possible range 0-10), both used as tests of verbal memory and learning; (3) verbal fluency (animal naming) used as a measure of language and executive function, and (4) processing speed measured by timed letter cancellation test (possible range 0-65).

### Assessment of Oxidative Stress Biomarkers in Serum

The selection of biomarkers was based on their suitability as indicators of antioxidant status in large-scale studies [14]. Serum samples were analyzed in 2012-2013 after being stored in freezers at −80°C for 3-10 years; all biomarkers analyzed were shown to have adequate long-term stability under these conditions [18,19]. Biomarkers were determined using an autoanalyzer (LX20-Pro, Beckman-Coulter, Woerden, The Netherlands). BAP and d-ROM kits were obtained from Diacron Labs (Diacron International s.r.l., 2016, Grosseto, Italy). TTL was obtained from Rel Assay Diagnostics (2016, Gaziantep, Turkey).

Concentration of d-ROMs was used as an index for the production of ROS, with high values indicating higher oxidative stress. The d-ROMs assay measures the hydroperoxide concentration based on the principle that the amount of organic hydroperoxides present in serum is related to the levels of free radicals from which they are formed [20,21,22]. The results of the assay are expressed in Carratelli units (U.CARR), where one U.CARR corresponds to 0.8 mg/L of hydrogen peroxide (H_2_O_2_). BAP reflects the total antioxidant capacity of serum. The BAP assay, expressed in meq/L, is a simple photometric test which measures the concentration of total antioxidants by their capacity to reduce iron from the ferric to the ferrous form. TTL, expressed in μmol/L, was used as a marker of protein oxidation [23]. The number of free thiol groups as cysteine residues in proteins measures favorable redox status, and a low number of thiol groups is indicative of increased oxidative stress.

Biomarkers were assayed at different times in different centers (Czech towns in May 2012, Krakow in July 2013, and Kaunas in May 2013). To correct for potential shifts in the assays, data from control samples were used for calibration. Agreement of d-ROMs measurements between centers was high, and no correction was applied. Observed shifts in BAP and TTL assay results were corrected by multiplying them by a relevant factor (0.83 and 0.86 for BAP, and 1.26 and 1.46 for TTL in Czech towns and Kaunas, respectively), with assay results for Krakow used as the standard.

### Covariate Assessment

We included the following potential covariates in the statistical analysis: age at cognitive assessment, sex, study center, case-control status, education (primary or less, secondary, and tertiary), current socioeconomic status using employment status (employed, self-employed, retired, retired but still working, unemployed, and other), smoking status (never, current, and former), average alcohol intake in the past year expressed in grams per day (0, <5, 5-20, and >20 g in women and 0, <10, 10-50, and >50 g in men), and depressive symptoms measured by CES-D 20-item scale [24] (yes vs. no using a score of 16 as the threshold). We also included self-reported history of major chronic conditions: MI, stroke, hypertension, and diabetes (all yes vs. no).

### Statistical Analysis

To allow comparison between tests, cognitive test results were standardized to z-scores (mean = 0; standard deviation [SD] = 1) using the full study sample means [17]. In addition, a global cognitive score was derived by averaging the standardized scores on each of the 4 tests. For all cognitive tests as well as the global cognitive score, higher scores indicate better cognitive performance. Levels of d-ROMs and BAP were categorized according to the manufacturer's instructions. Levels of d-ROMs were categorized into not increased (≤340 U.CARR), moderate (341-400 U.CARR), and high oxidative stress (>400 U.CARR). BAP status was categorized as deficiency (<2,000 meq/L), borderline deficiency (2000-2,200 meq/L), and normal values (>2,200 meq/L). There are currently no established clinical cutoffs for TTL, leading us to quartile them in the analysis with the bottom quartile (25%) taken to indicate higher oxidative stress. Cross-sectional associations of established risk factors for cognitive function with oxidative stress markers were assessed using linear regression, with adjustment for age, sex, center, and case-control status.

Two sets of analyses were conducted to estimate cross-sectional and prospective associations of oxidative stress markers with cognitive performance. All variables used in cross-sectional analysis were measured at baseline. In prospective analyses, we used biomarkers and covariates from baseline and cognitive function measures from follow-up. As data at follow-up were not collected in Kaunas, only 2 cohorts (Czech towns and Krakow) were included in the prospective analysis, where cognitive performance was assessed on average 3.7 ± 0.4 years (minimum = 1.8 years; maximum = 5.5 years) later.

In both cross-sectional and prospective analyses, linear regression was used to model the association of biomarkers (in categories) and cognitive z-scores. To test for linear trend across successive biomarker categories, we used biomarkers as ordinal variables in linear regression. We repeated the analysis with biomarkers modelled as continuous variables and tested for nonlinearity by including quadratic terms for biomarkers in the regression equations.

As data come from a nested case-control design originally set up to study cardiovascular disease and mortality, all regression models were adjusted for case-control status, history of MI or stroke, and matching criteria (sex, age group, and study center). The models were then additionally adjusted for education, socioeconomic status, health behaviors, and depression. Finally, we also adjusted the models for history of diabetes and hypertension. All covariates were modelled as categorical variables (categories shown in Table 1).

Prospective models (cognition from follow-up) were additionally adjusted for cognitive testing occasion (first test vs. re-test) to control for possible learning effects from repeated test taking. We also controlled for follow-up time in prospective models and tested for interactions between biomarkers and the main demographic variables (sex, age, and study center). In order to facilitate interpretation of the regression coefficients associated with biomarkers, we compared them with the effect of age on cognition by dividing the regression coefficients by the effect of 1-year increase in age on cognition.

From 6,358 participants included in the nested case-control study, those with incomplete oxidative stress (*n* = 49) or cognitive data (baseline *n* = 1,973; follow-up *n* = 3,384) were excluded from the analysis (see online suppl. Table S1; for all online suppl. material, see www.karger.com/doi/10.1159/000450702). The proportion of missing data of baseline covariates was generally low and ranged from 0.3 to 2.5% per covariate. Missing data on covariates at baseline were replaced using multiple imputation by chained equations [25] and using all available data for all variables in the analysis to generate 10 imputed datasets. After excluding observations with missing data on oxidative stress and cognition, there were 4,304 participants available for cross-sectional and 2,882 participants for prospective analysis.

We conducted a number of sensitivity analyses. First, we restricted the analysis to controls in order to ensure that the results were not driven by cases, including persons who had died or had cardiovascular disease. Second, we excluded participants who died within 2 years of the baseline survey, suffered a nonfatal MI or stroke during follow-up, or had existing hypertension or diabetes at baseline. Finally, we conducted an additional complete case analysis in the subset of participants with repeated cognitive measures (*n* = 1,540), adjusting for baseline cognitive function and also including an interaction term between age and baseline cognitive status. All analyses were conducted in Stata 14 (Stata Statistical Software, StataCorp LP, release 14, 2015, College Station, TX, USA).

### Statement of Ethics

The study was approved by the ethics committees at University College London and University College Hospital and the local ethics committees in each participating center. Written informed consent was obtained from all participants.

## Results

Descriptive characteristics of cases and controls are shown in Table 1. Around two thirds of participants were male, due to data for the current analyses being drawn from a case-control design where men were more likely to be selected due to higher mortality and cardiovascular disease rates. Average baseline age of participants was 63.9 years, and age was inversely associated with cognitive performance; in cross-sectional analysis, 1-year increase in age was associated with −0.03 SD (95% confidence interval [CI] −0.04, −0.03) lower scores on verbal fluency, −0.04 SD (95% CI −0.05, −0.04) lower scores on immediate recall, and −0.04 SD (95% CI −0.04, −0.03) lower scores on delayed recall, processing speed, and global cognition.

In cross-sectional analyses, d-ROM levels were significantly inversely associated with verbal fluency and the global cognitive score (Table 2). The differences in test scores between the highest and lowest categories of d-ROMs correspond to an age effect of roughly 3 years for verbal fluency and 1.5 years for global cognition. BAP was not related to performance on any of the cognitive tests. Compared to those in the top quartile, participants in the bottom quartile of TTL had significantly lower scores on immediate and delayed recall tests.

**Fig. 1:**
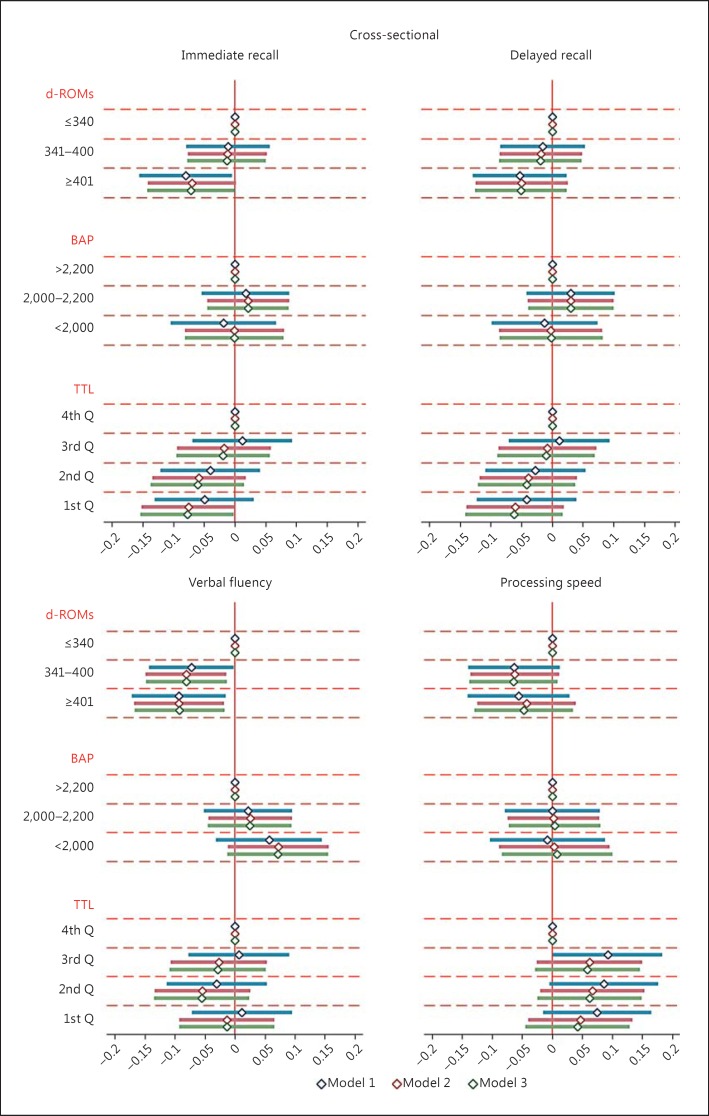
Cross-sectional associations of oxidative stress markers with cognitive scores. Data shown are unstandardized regression coefficients (diamonds) with 95% CIs (solid lines). Model 1 (1st solid line) is adjusted for age group, sex, center, case-control status, and cognitive test versus retest status. Model 2 (2nd solid line) is additionally adjusted for education, socioeconomic status, smoking, alcohol, depression, history of cardiovascular disease, and stroke. Model 3 (3rd solid line) is additionally adjusted for history of diabetes and hypertension. Biomarker units: d-ROMs, U.CARR; BAP, meq/L; and TTL, μmol/L. BAP, biological antioxidant potential; d-ROMs, derivatives of reactive oxygen metabolites; Q, quartile; TTL, total thiol level; U.CARR, Carratelli units.

**Fig. 2:**
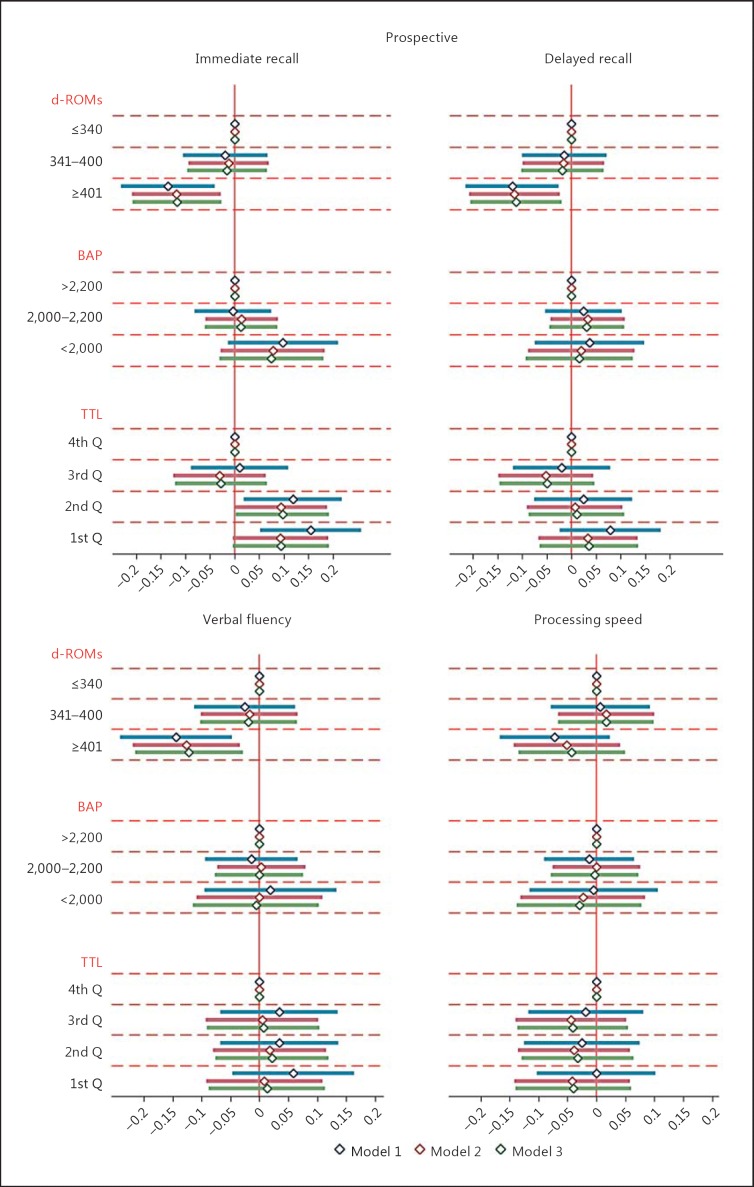
Prospective associations of oxidative stress markers with cognitive scores. Data shown are unstandardized regression coefficients (diamonds) with 95% CIs (solid lines). Model 1 (1st solid line) is adjusted for age group, sex, center, case-control status, and cognitive test versus retest status. Model 2 (2nd solid line) is additionally adjusted for education, socioeconomic status, smoking, alcohol, depression, history of cardiovascular disease, and stroke. Model 3 (3rd solid line) is additionally adjusted for history of diabetes and hypertension. Biomarker units: d-ROMs, U.CARR; BAP, meq/L; and TTL, μmol/L. BAP, biological antioxidant potential; d-ROMs, derivatives of reactive oxygen metabolites; Q, quartile; TTL, total thiol level; U.CARR, Carratelli units.

In prospective analysis (Table 2), high baseline d-ROM levels continued to be inversely associated with verbal fluency and the global cognition score at follow-up. High baseline d-ROM levels were also inversely associated with immediate and delayed recall at follow-up. Comparing with the effect of age, the differences between the highest and lowest categories of d-ROMs were equivalent to roughly 4 years for verbal fluency and 2.5 years for global cognition. There were no associations between baseline BAP deficiency and any of the cognitive domains or global scores at follow-up. In contrast, the prospective association of TTL with immediate recall was statistically significant but in the opposite direction to that in cross-sectional analyses. After additional adjustment for history of diabetes and hypertension, the associations remained relatively unchanged (Fig. 1, 2; online suppl. Fig. S1 for global cognition).

Results from linear regression using biomarkers as continuous variables were not statistically significant, with the exception of prospective models for d-ROMs and verbal fluency (*p* = 0.017) and TTL and immediate recall (*p* = 0.018). The models were not significantly improved by adding quadratic terms for biomarkers to account for possible nonlinearity. We found no significant interactions between biomarkers and study center.

Sensitivity analyses suggested that results for the observed significant associations of d-ROMs and TTL with cognitive performance measures were mostly robust (see online suppl. Tables S2-S5). However, the prospective associations of increased d-ROM levels with verbal fluency and global cognition were attenuated and no longer significant in an analysis restricted to controls (see online suppl. Table S2). In a complete case analysis in the subset of participants with repeated cognitive measures, adjusting for baseline cognition did not appreciably change the pattern of results. The inclusion of an interaction term between baseline cognition and age also made no difference in the results. Overall, the associations of d-ROMs with verbal fluency and global cognition and of TTL with word recall were weaker in complete case analyses compared to analyses using multiply imputed data, possibly reflecting the smaller numbers of observations.

## Discussion

To the best of our knowledge, this study is the first investigation of serum oxidative stress markers in relation to domain-specific cognitive performance in a large general population sample of older adults. We found modest associations between oxidative stress markers, d-ROMs in particular, and cognitive function in early old age. Increased levels of d-ROMs were associated with poorer verbal fluency and global cognition both at baseline and at 3-year follow-up. TTL was associated with immediate recall, but the pattern of cross-sectional results contradicted prospective associations. We observed no association between BAP status and cognitive performance.

Strengths of this study include the objective assessment of serum antioxidant status using a combination of biomarkers, assessment of cognitive function by repeated administration of neuropsychological tests, large well-characterized samples from 3 Central and Eastern European populations, and an extensive assessment of major risk factors for cognitive function allowing for control of confounding.

Several limitations of this study should be mentioned. First, the response rate to the baseline survey was relatively low, although similar to most contemporary population-based studies [26]. Responders were more likely to be healthier and socially advantaged than nonresponders [16]; therefore, slightly healthier and socially advantaged individuals were probably overrepresented in our sample. However, we would not expect response rates to bias the estimates of the associations between biomarkers and cognition. The second limitation relates to missing data. The prospective sample was restricted to participants with cognitive function at follow-up, and attrition appeared to be higher in participants with lower baseline cognitive scores. Thus, loss to follow-up could have affected our prospective results. As a consequence, potential confounding and bias cannot be definitively ruled out, and causal inferences can only be made with caution. Third, because the analytical sample was selected in a nested case-control design, the overrepresentation of cases with an increased likelihood of dying or dropping out before follow-up is a concern. We included the variables which were used to select participants into the case-control study as covariates in the analysis, and this approach should produce unbiased estimates. The results of sensitivity analysis restricted to controls were also broadly similar to the results of the main analysis, although the prospective results for d-ROMs and cognitive function were somewhat weaker than in the main analyses (see online suppl. Table S2). Fourth, biomarker measurements were not repeated at follow-up. However, we have demonstrated that associations of d-ROM levels with performance in specific cognitive domains remained statistically significant 3 years after baseline. In addition, we used only 2 repeated measurements of cognitive performance collected over a relatively short interval, which precluded us from studying decline in cognitive performance over time. Finally, our measure of global cognition, although less likely to be affected by measurement error than individual tests, has limited coverage as it reflects the tests used to derive it.

We are not aware of other studies to date which have assessed the association of oxidative stress markers with cognitive performance in the general population. An early community study in 1,166 participants aged 60-70 years from Nantes (France) found an increased risk of decline in MMSE scores over a 4-year period in persons with high baseline levels of thiobarbituric acid-reactive substances, an indicator of free radical-induced lipid peroxidation [10]. A dose-response effect was also reported between the biomarker levels and odds of cognitive decline. In our study, high d-ROM levels were also associated with worse global cognition at follow-up and showed a dose-response effect. However, our global cognitive score had restricted domain coverage, and we used a different biomarker in a younger sample. Therefore, direct comparisons require caution.

In our study, increased d-ROM levels were inversely associated with verbal fluency and global cognition, both cross-sectionally and at 3-year follow-up, and there was evidence for a linear trend. High d-ROM levels were also prospectively associated with worse immediate and delayed recall. Poorer performance on verbal fluency in participants with increased d-ROM levels corresponded to an age effect of roughly 3 years in cross-sectional and 4 years in prospective analyses. Age effects for global cognition were roughly 4 years, taking together both cross-sectional and prospective analyses. These effects are not negligible and, given the imprecision in measurement of cognition and the probable biological variations in biomarker concentrations, it is likely that the associations were underestimated.

One possible mechanism through which oxidative stress markers, such as d-ROMs, could affect cognitive function is vascular risk factors. The role of oxygen free radicals and other ROS is considered important in the development of atherosclerosis, stroke, and other cardiovascular diseases [27,28]; these conditions are likely to act as risk factors for cognitive decline or dementia [29,30]. In addition, there is growing evidence that oxidative stress is involved in the pathogenesis of both AD and vascular dementia [31,32]. Recently, there has also been growing recognition that insulin production and signaling are severely impaired in the AD brain, resulting in impaired glucose metabolism and mitochondrial function and, in turn, increased production of ROS [33]. However, in this study, adjusting for or excluding participants with underlying cardiovascular conditions or diabetes did not appear to explain the observed associations between d-ROMs and cognitive performance measures.

Levels of d-ROMs were not associated with processing speed or with memory in cross-sectional models, although the direction of the association was the same for all cognitive tests and differences in the strength of the observed associations may also reflect differences in measurement precision [34]. It is possible that verbal fluency, a measure of executive function, is especially sensitive to age-associated changes and disease processes. Future studies should use more specific tests of executive function to evaluate the association with blood-based oxidative stress markers.

TTL was associated with immediate recall, but the pattern of cross-sectional and prospective associations was in opposite directions. One possible explanation is model misspecification; this could include an omitted variable, selection bias, regression to the mean, and specification or measurement error. However, having performed various checks [35], the results did not change substantially. Thiols are a major constituent of the body's total antioxidants, play an important role in the defense against ROS, and their concentrations have been shown to be decreased in numerous diseases including cardiovascular disease and diabetes [23]. We are not sure what can explain our inconsistent results.

In the present study, BAP showed no relation with cognitive performance. In a recent cross-sectional study of 2,518 German older persons, BAP also showed inconsistent associations with frailty [36], a marker for future cognitive impairment, which is associated with executive function [37].

In this study, d-ROMs emerged as a more promising marker for cognitive aging than BAP or, less consistently, TTL. Previously, d-ROMs and TTL were shown to be associated with all-cause and cardiovascular mortality in a joint analysis of the current cohorts and a German cohort [13]. Blood-based biomarkers could provide cost- and time-effective means of identifying groups at risk of cognitive decline. For example, results from a recent effort to construct and validate a risk index for cognitive decline based on blood-based markers were moderately positive. The index, including 8 markers which had previously been shown to be associated with cognitive aging, was modestly predictive of 11-year decline on the MMSE after controlling for age and baseline cognition [38]. Findings of our study, if successfully replicated, could be useful to extend such biomarker scores.

## Disclosure Statement

The authors report no conflicts of interest.

## Figures and Tables

**Table 1:** Descriptive characteristics of the study sample by case-control status in participants with complete observations

	Cross-sectional			Prospective		
	cases (*n* = 1,177)	controls (*n* = 2,901)	*p*	cases (*n* = 467)	controls (*n* = 2,250)	*p*
Immediate recall	19.7 (4.3)	20.8 (4.0)	<0.001	20.9 (4.3)	21.6 (4.0)	0.001
Delayed recall	6.8 (2.0)	7.2 (1.9)	<0.001	7.0 (2.0)	7.2 (1.9)	0.020
Verbal fluency	19.9 (6.4)	21.2 (6.4)	<0.001	21.8 (6.7)	22.7 (6.4)	0.012
Letter cancellation	15.4 (5.5)	16.7 (5.0)	<0.001	16.6 (4.9)	17.5 (4.7)	<0.001
d-ROMs, U.CARR	386.6 (86.1)	365.8 (73.9)	<0.001	381.5 (85.4)	368.0 (83.1)	0.002
d-ROM categories			<0.001			0.001
<340 U.CARR	28.5	35.9		31.7	35.7	
341–400 U.CARR	32.9	35.6		28.9	34.0	
>400 U.CARR	38.7	28.5		39.4	30.3	
BAP, meq/L	2,144.2 (194.3)	2,129.1 (194.8)	0.024	2,188.5 (195.2)	2,196.7 (201.5)	0.410
BAP categories			0.089			0.856
>2,200 meq/L	33.7	31.1		46.0	45.6	
2,000–2,200 meq/L	44.1	43.8		41.8	41.2	
<2,000 meq/L	22.2	25.1		12.2	13.2	
TTL, μmol/L	489.5 (90.5)	493.4 (83.7)	0.199	516.8 (86.0)	516.5 (81.5)	0.947
TTL categories			0.869			0.852
4th Q	22.4	23.0		24.0	24.1	
3rd Q	24.6	24.8		26.3	25.0	
2nd Q	25.1	25.6		24.2	26.0	
1st Q	27.9	26.6		25.5	24.9	

Age group			0.724			0.242
<50 years	3.0	3.4		1.1	1.9	
50 to <55 years	6.1	5.1		10.9	10.1	
55 to <60 years	12.6	13.0		15.1	14.1	
60 to <65 years	33.5	33.0		23.6	19.6	
65 to <70 years	36.8	38.2		28.3	32.2	
>70 years	8.0	7.4		20.9	22.0	
Center			0.493			0.033
Czech towns	30.4	32.1		55.7	50.3	
Krakow	27.3	26.8		44.3	49.7	
Kaunas	42.3	41.1		n.a.	n.a.	
Male	65.9	66.6	0.676	66.8	67.7	0.712
Education			<0.001			0.002
Primary or less	15.7	10.5		10.1	8.7	
Secondary	57.3	54.0		71.1	64.6	
College or university	26.9	35.4		18.8	26.7	

SES			<0.001			0.031
Employed	12.1	15.6		22.3	22.6	
Self-employed	1.5	2.3		3.2	5.3	
Retired, still working	11.2	17.3		7.9	11.3	
Fully retired	70.2	61.9		62.5	57.5	
Unemployed	2.0	1.0		3.2	2.0	
Other	2.9	1.8		0.9	1.3	
Smoking status			<0.001			<0.001
Never smoker	37.3	50.2		32.8	42.1	
Former smoker	31.4	30.9		31.5	34.8	
Current smoker	31.4	18.9		35.8	23.1	
MI	16.1	9.1	<0.001	10.1	8.1	0.173
Stroke	5.4	3.9	0.042	2.8	2.9	0.861
Hypertension	63.6	59.1	0.008	62.1	52.8	<0.001
Diabetes	20.0	11.7	<0.001	20.1	12.8	<0.001
Alcohol intake, g/day			<0.001			0.145
None	21.6	15.5		21.8	19.6	
Low	58.0	60.7		57.4	56.1	
Medium	16.8	21.1		16.3	20.6	
High	3.6	2.6		4.5	3.7	
Depressive symptoms	29.6	21.7	<0.001	29.1	21.4	<0.001

Means and standard deviations in parentheses are presented for continuous variables and proportions for categorical variables. *p* values from two-tailed *t* tests comparing means or χ^2^ tests of independence between cases and controls. BAP, biological antioxidant potential; d-ROMs, derivatives of reactive oxygen metabolites; MI, myocardial infarction; n.a., not applicable; Q, quartile; U.CARR, Carratelli units; SES, socioeconomic status; TTL, total thiol level.

**Table 2:** Cross-sectional and prospective associations of d-ROM status, BAP status, and TTL with cognitive performance z-scores

	Immediate recall z-score, β (95% CI)	Delayed recall z-score, β (95% CI)	Verbal fluency z-score, β (95% CI)	Letter cancellation z-score, β (95% CI)	Global cognition, β (95% CI)
*Cross-sectional (n = 4,304)*					
d-ROMs					
≤340 U.CARR	0.00	0.00	0.00	0.00	0.00
341–400 U.CARR	–0.01 (–0.08, 0.05)	–0.02 (–0.09, 0.05)	–0.08 (–0.15, −0.01)*	–0.06 (–0.14, 0.01)	–0.04 (–0.09, 0.00)
≥401 U.CARR	–0.07 (–0.14, 0.00)	–0.05 (–0.12, 0.03)	–0.09 (–0.17, −0.02)*	–0.04 (–0.13, 0.04)	–0.06 (–0.12, −0.01)*
*p* for trend	0.055	0.184	0.012	0.267	0.016
BAP				
>2,200 meq/L	0.00	0.00	0.00	0.00	0.00
2,000–2,200 meq/L	0.02 (–0.04, 0.09)	0.03 (–0.04, 0.10)	0.03 (–0.04, 0.10)	0.00 (–0.07, 0.08)	0.02 (–0.03, 0.07)
<2,000 meq/L	–0.00 (–0.08, 0.08)	–0.00 (–0.08, 0.08)	0.07 (–0.01, 0.16)	0.00 (–0.09, 0.10)	0.02 (–0.04, 0.08)
*p* for trend	0.967	0.987	0.088	0.902	0.506
TTL					
4th Q	0.00	0.00	0.00	0.00	0.00
3rd Q	–0.02 (–0.10, 0.05)	–0.01 (–0.09, 0.07)	–0.03 (–0.11, 0.05)	0.06 (–0.03, 0.14)	0.00 (–0.06, 0.06)
2^nd^ Q	–0.06 (–0.14, 0.01)	–0.04 (–0.12, 0.04)	–0.06 (–0.14, 0.02)	0.06 (–0.02, 0.15)	–0.02 (–0.08, 0.03)
1st Q	–0.08 (–0.15, −0.00)*	–0.06 (–0.14, 0.02)	–0.02 (–0.09, 0.06)	0.04 (–0.04, 0.13)	–0.03 (–0.09, 0.03)
*p* for trend	0.023	0.081	0.619	0.364	0.233

*Prospective (n = 2,882)*					
d-ROMs					
≤340 U.CARR	0.00	0.00	0.00	0.00	0.00
341–400 U.CARR	–0.01 (–0.09, 0.07)	–0.01 (–0.10, 0.07)	–0.02 (–0.10, 0.07)	0.02 (–0.06, 0.10)	–0.01 (–0.07, 0.05)
≥401 U.CARR	–0.12 (–0.21, −0.03)*	–0.1 (–0.21, −0.02)*	–0.12 (–0.22, −0.03)^**^	–0.05 (–0.14, 0.05)	–0.10 (–0.17, −0.03)^**^
*p* for trend	0.012	0.018	0.011	0.360	0.004
BAP					
>2,200 meq/L	0.00	0.00	0.00	0.00	0.00
2,000–2,200 meq/L	0.01 (–0.06, 0.09)	0.03 (–0.04, 0.11)	–0.00 (–0.08, 0.08)	–0.00 (–0.08, 0.07)	0.01 (–0.04, 0.07)
<2,000 meq/L	0.08 (–0.03, 0.18)	0.02 (–0.09, 0.13)	–0.00 (–0.11, 0.11)	–0.02 (0.13, 0.08)	0.02 (–0.06, 0.10)
*p* for trend	0.188	0.550	0.973	0.730	0.602
TTL					
4th Q	0.00	0.00	0.00	0.00	0.00
3rd Q	–0.02 (–0.12, 0.07)	–0.05 (0.14, 0.05)	0.01 (–0.09, 0.11)	–0.04 (–0.13, 0.06)	–0.02 (–0.09, 0.04)
2nd Q	0.10 (0.00, 0.19)	0.01 (–0.09, 0.11)	0.02 (–0.08, 0.12)	–0.04 (–0.13, 0.06)	0.02 (–0.05, 0.09)
1st Q	0.10 (0.00, 0.20)	0.04 (–0.06, 0.14)	0.02 (–0.08, 0.12)	–0.03 (0.13, 0.07)	0.03 (–0.04, 0.10)
*p* for trend	0.009	0.305	0.761	0.456	0.278

Models are adjusted for age group, sex, study center, education, socioeconomic status, smoking, alcohol, depression, cardiovascular disease history and case-control status. Prospective models are additionally adjusted for cognitive test versus retest status. *p* for trend calculated by using biomarker categories as an ordinal variable in linear regression. BAP, biological antioxidant potential; CI, confidence interval; d-ROMs, derivatives of reactive oxygen metabolites; Q, quartile; TTL, total thiol level; U.CARR, Carratelli units.

* *p* < 0.05;

** *p* < 0.01.
